# History of Medicine

**Published:** 1873-07-01

**Authors:** Lyman Ware

**Affiliations:** Chicago


### HISTORY OF MEDICINE.

AN ADDRESS DELIVERED BEFORE THE ALUMNI ASSOCIATION OF CHICAGO MEDICAL COLLEGE, BY LYMAN WARE, M.D., OF CHICAGO.

As it is my privilege and great pleasure, gentlemen, to offer, according to our established custom, some subject for your consideration, upon retiring from the chair, I have chosen as that subject, History of Medicine, which has been fascinating to me, and I hope may not be uninteresting to you.

It is well for us to seriously consider whether medicine is walking hand in hand in progress with her sister arts of utility, and wherein we can add our mite toward the advancement of our growing art—as it behooves us in these days of progress and of action to look well that our duty is discharged with promptness and fidelity.

For centuries medicine was not a science, but an art, and was designated as the "Art of Healing." No nation or tribe, civilized or barbarous, is known that has not had some species or other of medicine, learned or ignorant, natural or superstitious, which has ever aimed to promote health and alleviate human suffering, however different may have been the means employed. Probably the first step toward the foundation of medicine was in the form of diet. It was first observed that in certain indispositions a diminution of food, which was coarse and crude, tended toward recovery; subsequently the quality as well as the quantity was changed, and liquid food was given. Thus, as each patriarch overlooked the affairs of his own household, in time he became familiar with the regulation of diet and administration of simple remedies or decoctions of various herbs ; and thus, soon was established the first dogma in medicine.

Every kind of medication which has cured one disease must also cure all analogous diseases. The inhabitants of earth early acquired the conviction that nature alone was insufficient in all cases to aid them, and that God helps most those who help themselves.

A little reflection easily explains why their temples were transformed into dispensaries, where patients from the most distant countries came to receive counsel and treatment. It was an age of ignorance, credulity and superstition. Diseases were regarded wholly as a celestial chastisement, and an evidence of the Divine wrath or displeasure, or the result of some malign influence, rather than as the effect of natural causes; and pestilences, which ravaged the nations and frequently almost depopulated towns and cities, were unhesitatingly referred to some unpropitious conjunction of the planets, or to the machinations of the devil. Therefore it was deemed more advisable to resort to prayers, expiations, sacrifices and exorcisms than to medicine. However strange, it is nevertheless true, the same general idea regarding the cause of disease is more or less prevalent today — as was so clearly manifested by the great outcry all over the land on account of the so-called materialistic proposition of Sir Henry Thomson.

As Egypt, during these earliest times, was pre-eminently in advance of all other nations in literature and the fine arts, so was she in medicine. As the Romans drew from the Greeks, so drew the Greeks from the Egyptians. Probably the first account of man practicing the art of medicine as a profession is that given in Genesis—nearly two thousand years before Christ — where Joseph commands the physicians to embalm his father. There are but few early biblical references to physicians; yet it is hardly possible that any antagonism could have existed between them and the prophets, notwithstanding the account given in Chronicles of Ara, who it is said "sought not the Lord, but the physicians;" immediately adding, either by way of a little humor or sarcasm, as if it served him well, "and Ara rested with his fathers."

There is so much of mythology intermingled in the accounts of the early history of medicine, that it is difficult always to separate the true from the false—the man from the gods. Chirou, the Centaur, had his office or den in a cave, and counted for his pupils all the celebrities of the age. Among the foremost was our father Æsculapius, of whom the god of the infernal regions complained to Jupiter that he rescued too many of his victims; and for such audacity earth suddenly knew him no more. Remembering his sad end, it is sarcastically said, his sons refrain from like prodigies.

The keenest observer, cleverest writer of early medicine, and the author of the "Reign of Medical Law," was undoubtedly Hippocrates. So high was the veneration in which he was held, that for centuries no one ventured to hold opinions contrary to his, or to penetrate deeper into the mysteries of health and disease. Born 500 years before the Christian era, of a family in which the practice of medicine was hereditary—the paternal side looking back to Æsculapius, the maternal to Hercules—he combined all the qualities that go to make a successful and worthy practitioner — intelligence, sincerity, love of his art and humanity. To him we are greatly indebted for that foundation upon which so much has been built, is building, and yet remains to be built. It was he who enunciated the dogma that "Diseases are cured by their contraries," a doctrine that held undisputed sway for more than twenty centuries, and when its converse was proclaimed to mankind, gave rise to much and bitter controversy. To-day we hold the empty theories of both in equal contempt. What care we how disease is cured — whether by sugar of milk or calomel, by fire or water, by perfect rest or constant movement? The alleviation of human suffering should ever be our highest aim, and not the confirmation of pet theories.

From the earliest ages down to the present time there has ever been an intense desire, with a view either to wealth or fame, to discover one remedy or law which shall prove of universal application. Sometimes a fancied discovery of this kind is called a doctrine, a method, a treatment, or a cure, according as it suits the fancy of the originator. All these, or the majority of them, true perhaps to a limited extent—and because true, so far successful — have failed, and always will fail when indiscriminately or universally applied. We believe in no panacea. No discovery of this kind can, or ever will be made, simply because there is no such remedy or law to discover. Disease, like every other phenomena, is subject to an infinity of laws, which must be met according to the form and direction which in each case they may have assumed.

While little attention was paid to so practical a subject as anatomy, the mind was ever busy concocting and discussing theories. The collection of Hippocratic aphorisms formed the subject of discussion and controversy for centuries. The most important aphorisms were those of coction and crisis, and the theory of the four elements and the four humors.

It was under the reign of the Ptolemies that the art of medicine took such rapid strides toward perfection. They encouraged not only the fine arts and general literature, by the establishment of the famous library of 60,000 volumes, but also favored dissections, by giving over to the scalpel all criminals and those who incurred public expense. To mitigate public opinion and prejudice against thus obtaining anatomical knowledge, the Ptolemies themselves participated in these dissections, and joined in anatomical discussions. Perhaps it may not be unkind to compare the praiseworthy action of these heathen rulers, who were surrounded by superstition and prejudice of every kind, with our own wise, liberal legislators, who most worthily and consistently refuse, unpaid, not only to favor the study of anatomy, which they have caused to be a penitentiary offence, but willingly do they exact large fines from those unhappy subjects who by hook or by crook, or rather by theft and concealment, have not obtained a most thorough knowledge of this important branch of their profession. Indeed, "Truth is stranger than fiction;" and as generations in the future read of our Christian and humane law-givers, they will doubt the truth of history.

When Alexandria was ruthlessly destroyed, and with it the library of the world, so perished for ages liberal and progressive medicine. Doubtless this retardation was not owing wholly to the abandonment of the study of anatomy, but also to the rapid extension of Christianity, which engendered in every mind a passion for religious controversy, to the exclusion of everything medical, disorganized the schools, and discredited the *profane* sciences. Everything was swallowed up in the bottomless abyss of scholasticism and demonology. Wars, pestilence and famine were the order of the day. As God was invoked in vain, men turned to Satan. They wasted their time and energies in vain and fruitless discussions, such as whether a spirit could live in a vacuum; and, in that case, would the vacuum be complete? Whether Adam and Eve, not being born in the natural way, showed the umbilical. They theorized concerning vital principles, and other mysterious entities; heaped hypotheses on hypotheses, careless of their foundation.

Fortunately, it was at this time the prevailing opinion that there was an antagonism between practice and theory, experience and reason. The practitioner must be confined to observation, the theorist to logic. In this way mankind was not made the victim of visionary men. Perhaps it is to be regretted that so salutary an opinion should have ever been disproved. Certain it is, had it been, Haunkernaun would have been unknown to fame.

The Church, whether Roman or Protestant, ever looked with jealousy toward innovation and investigation. During these days of darkness and power she punished terribly all those who ventured to even question the authority of the age. It is terrible to think how many great lights must have been extinguished — how many discoveries nipped in the bud by the vigorous stamping out of heresy. Such was the sad end of Michael Servitus, the discoverer of pulmonary circulation, in the middle of the sixteenth century, almost anticipating by fifty years the great discovery of Harvey; and for his pains, Calvin burned him and his works together at the stake, leaving but one scorched volume to show what he had done; and he was only one of five hundred that were burned at Geneva alone. When Eddin, a doctor of Sarboum, remonstrated against such wholesale cruelty, for his philanthrophy he was shut up between four walls, without food or drink.

While such a belief in demonism prevailed everywhere, it is not to be wondered that the utility of medicine was doubted. The efficacy of relics and charms was universally acknowledged. The efforts of physicians were directed to the invention of nostrums and counter-charms — not to the investigation of the causes of disease, the careful observation of their phenomena, or the mode of action of the remedies prescribed for them. Even Servitus, when he had almost grasped the great secret of the complete circulation of the blood, instead of proceeding with the investigation before the little bonfire of Calvin was lighted, assumed all other errors except the one he had disproved and discredited.

In 1514 a man of genius and courage, a true reformer, Tesalius, made his appearance. He it was who did not see Galius' porosities of the heart, and dared say so.

It is interesting to read Harvey's own words of his truly wonderful discovery: "I floated undecided, without knowing upon what opinion to rest. Finally, from redoubled care, and by comparing the various results, I believed I had put my finger upon the truth, and commenced unravelling the labyrinth. From that time I never ceased to communicate my views, either to my friends or to the public." And yet it was a half century before his discovery was acknowledged. During these times—the middle ages—there prevailed the terrible doctrine that attributed everything to the agency of evil spirits, and medicine became a routine of superstitious practices. We are told of one who practiced blood-letting to an unlimited extent. After three bleedings he would add a fourth, for the reason that there are four seasons, four quarters of the globe, and four cardinal points. After the fourth he took a fifth, because there are five fingers in the hand. To the fifth he would add a sixth, for did not God create the world in six days. But the number must be made seven, there being seven days in the week and seven sages of Greece. An eighth bleeding had to follow, eight being a round number; and a ninth, because God loves odd numbers. Such and such a plant was considered beneficial if gathered at the new moon, but deadly poison if gathered at the moon wane. If the solar eclipse took place, a dragon was supposed to have swallowed the sun. When a pestilence raged, the invisible arrows of an offended Deity struck down the victims; when a volcano burst forth, some subterraneous demon was presumed to be at work. Even the Reformer, Luther, believed that the devil tormented him with an earache.

Within the past few years the same idea or spirit was manifested by Brigham Young, when he said: "If we have faith to feel that the issues of life and death are in our power, we can say to disease, that is, the devil, 'Be ye rebuked in the name of Jesus, and let life and health come into the system of this individual from God to counteract this disease,' and it will be done."

From this state of affairs the transition to consider diseases as entities was most natural. To-day we speak of tumors being malignant or benign; of diseases as yielding or increasing, of attacking or invading, thus attributing to them powers of perception and action. The morbid entities which perceived, felt and acted, were the vindictive enemies of the human race. The term "heroic," as applied to practitioners and remedies, had its origin in this imaginary strife for life and death with diseases, as if they were intelligent and implacable foes to the existence of mankind. The attitude of the doctor toward disease was believed to be that of an antagonist, hence the epithet Allopathist, used by persons even of to-day, and by persons of education. If one declines being called an Allopathist, the chances are greatly in his favor of being stigmatized as some other "*ist*" which may be still more unpleasant.

The doctrine of signatures actually held sway until the beginning of the last century. It was a sweet and attractive idea to suggest that God had impressed a signature upon objects in nature capable of being used for cure, and that this signature consisted in a resemblance either to the disease to be healed or to the part where it was situated. The idea never can be forgotten as long as the Anglo-Saxon tongue is spoken. Under its guidance the sparkling eyebright was gathered by the mediæval oculist. Liverwort and lungwort were used to heal the organs they were considered to resemble. Jaundiced persons were immersed in baths of the yellow wild cucumber. Hæmorrhages were arrested after the application of the red-speckled blood-stone. Wine-colored gems were worn and called amethysts, because they performed the easy task of letting the wearer know when he had drunk too much (probably by an apparent haziness of outline of color). Scarlet bed-curtains were a cure for scarlet fever, measles, or any disease with a red eruption of the skin; and the grandfather of Maria Theresa died of small-pox, wrapped, by order of his physician, in twenty yards of scarlet broadcloth. The lung of a long-winded fox was a cure for asthma and shortness of breath; and the heart of a nightingale was prescribed for loss of memory. However beautiful and poetical the theory may have been, the practice was *nil.* As the proof of the pudding is in the eating, so is actual experience the test of all theories. Those which cannot stand this test are destined, sooner or later, to be thrown aside, even be their founder never so enthusiastic.

Hippocrates early pointed out the importance of ascertaining the temperature in disease; and yet more than two thousand years elapsed before any advancement was made in this direction. Nothwithstanding instruments of precisive and delicate workmanship have been provided, how few are there who avail themselves of this means of diagnosticating disease! Heat, from time immemorial, has ever been considered the principal phenomena of fever and acute disease; yet without a well-graduated thermometer, the determination of the amount of heat is mere guess-work. Wunderlich is the modern Hippocrates. By means of the thermometer he has fully confirmed the theories of the old man of Cos, of crisis and critical days. As we most devoutly wish positive diagnosis to transplant conjecture and imposture, as astronomy did astrology, as chemistry did alchemy, so must we avail ourselves of every means that determines definitely the extent of disease.

Before Franklin interfered in the monopoly of Jupiter, and claimed a seat in the ring of the gods, when the Æsculapian sons wished to impart electric life, it was customary to withdraw the fire from the electric eel.

Charlatans have long used this most valuable and powerful remedy as a panacea; but thanks to the untiring energies of a few hard-working, scientific men of our own time, the dark days of its history are over, and can never return. Though not a panacea, it is probably as useful as any other remedy in the *materia medica.* It is yet to be determined *when* and *how* this fiery agent can be best employed for the relief and mitigation of human suffering.

It is to Lœnnec the world is indebted for the science of auscultation; yet its appreciation was exceedingly limited for years after his death. Even as late '36 a writer spoke of the stetheoscope, sneeringly, as "The toy is a new toy;" as though the child would throw it aside the moment a newer one was granted. To-day auscultation, either intermediate or mediate, is universally acknowledged as absolutely necessary to the correct diagnosis of disease, and is one of the corner-stones of life insurance.

Percussion, aptly called the elder brother of auscultation, the difference in their ages being over half a century, had almost died at birth, and was disinterred by Coonivart, in 1808; but it was not really resuscitated until it became associated with auscultation.

The method of treating disease by hypodermic injections, which was suggested to Alexander Wood, of Edinburgh, by Ferguson's treatment of naevi by the injection of tincture of iron, is of still later date. It is to be regretted that it is not of more general use to-day. Many, became dissatisfied with the method on account of the exceedingly poor instruments that were at first used. So great indeed was the bias that a gentleman of high rank in the profession, only a short time since, denounced the instruments as worthless "*squirt-guns*," the use of which was unbecoming the dignity of the man or the physician.

As Allopathy strictly, so called, is now numbered in the past, where, we might well ask, is there a place to be found for Homœopathy? as her mission heretofore has ever been that of fault-finding and opposition, instead of tracing out the causes of and preventing disease. Of all the instruments which have ever yet been applied to scientific research, and the advancement of practical medicine, none certainly equal the microscope. It is, beyond a question, the most efficient means in the hands of the physician for the determination of disease; and he who neglects its aid, literally neglects a host of help.

It may not be unwise to consider for a moment what may be the medicine of the future. As many of the most valuable articles of the *materia medica* are of recent introduction, it is reasonable to suppose that others of at least equal value will be introduced. It is to be greatly hoped that the *modus operandi* of medicines will be better understood, thereby simplifying the treatment of many diseases. We well remember the wise words of a venerable practitioner who remarked that when he began his professional career he had twenty remedies for every disease; now he had one remedy for twenty diseases. The natural history of disease is exceedingly incomplete. One of the most eminent men in our land has said that here alone is ample scope for useful labor. A sure career of usefulness and honor is open to any who are able and willing to spend a series of years in first recording causes of disease, and, second, subjecting the accumulated histories to careful analysis and comparison.

The poet of the Greek race-course was right in saying, "'T is the trial that proves the man." Clinical researches and empirical decisions must always hold the most important part of any department of medicine. Never, probably, was there a time when they were so highly appreciated as now; so that with much confidence we can leave to be estimated by this measure the restorative system of therapeutics which has been so rapidly gaining ground during the past few years among all teachers of medicines.

Looking through the dim vista of the future, there are those who are enthusiastic enough to believe they catch a glimpse of that longed for Canaan when all curable diseases will be cured; preventable, prevented; and all contagious diseases, properly so-called, will be blotted out of existence. We stamp out the cattle-plague, and if the plagues of men touched the same obvious and immediate pecuniary interests as the cattle-plague, we might stamp out similar calamities among human beings.

